# Two Cases of *Legionella pneumophila* Pneumonia with Prolonged Neurologic Symptoms and Brain Hypoperfusion on Single-Photon Emission Computed Tomography

**DOI:** 10.1155/2016/5264681

**Published:** 2016-07-10

**Authors:** Hiromitsu Ohta, Susumu Yamazaki, You Miura, Akira Seto, Minoru Kanazawa, Makoto Nagata

**Affiliations:** ^1^Respiratory Medicine, Saitama Medical University, Morohongo 38, Iruma, Saitama 350-0495, Japan; ^2^Nuclear Medicine, International Medical Center, Saitama Medical University, 1397-1 Yamane, Hidaka, Saitama 350-0495, Japan

## Abstract

Cerebral and cerebellar symptoms are frequently associated with Legionnaires' disease. However, corresponding brain lesions are difficult to demonstrate using either computed tomography (CT) or magnetic resonance imaging (MRI). We report here two patients with* Legionella pneumophila* pneumonia accompanied by prolonged neurologic symptoms. In contrast to brain CT and MRI, which failed to detect any abnormalities, single-photon emission computed tomography (SPECT) showed multiple sites of hypoperfusion within the brains of both patients. These cases suggest that vasculopathy, which is detectable by SPECT, might be one of the causes of neurologic symptoms in patients with Legionnaires' disease.

## 1. Introduction

*Legionnaire pneumophila* is a common cause of community-acquired pneumonia and is associated with a high mortality rate. Legionnaires' infection demonstrates a variety of symptoms involving the respiratory, gastrointestinal, and neurologic systems. Symptoms of the central nervous system (CNS) are observed in approximately 40% of patients. Encephalopathy symptoms include altered or impaired consciousness, disorientation, and confusion [1, 2], whereas cerebellar symptoms include gait and limb ataxia [3]. Even in the presence of these symptoms, computed tomography (CT) scans of the brain often appear to be normal, and magnetic resonance imaging (MRI) seldom reveals brain lesions. In a previous case, Imai et al. reported that single-photon emission computed tomography (SPECT) using technetium-99m-hexamethyl-propylene amine oxime revealed brain lesions in a patient with Legionnaires' disease accompanied by CNS symptoms [4]. Here, we report our experience with 2 cases of* Legionnaire pneumophila* pneumonia with prolonged neurologic symptoms. In both cases, SPECT was useful in detecting brain lesions that were not observed by either CT or MRI.

## 2. Case Report

### 2.1. Case 1

A 60-year-old man was admitted to our hospital with the primary symptom of confusion. He lived in the dormitory of a factory that had a public bathhouse, in which bath water was circulated through a sterilizer system and reused. His medical history was unremarkable. He did not use any drugs but had a 60 pack-year history of smoking. Four days before admission, he had developed cough. On the day of admission (Day 1), his colleague noticed that he could not stand by himself and had slurred speech; thus, he brought him to the emergency department of our hospital.

On admission, he was restless and could not obey our orders. His temperature was 40.1°C, blood pressure was 154/82 mmHg, and pulse rate was 102 beats/minute. His oxygen saturation was 90% with oxygen supplementation at 4 L/minute using a facial mask. Pulmonary auscultation revealed coarse crackles in his right lung. Laboratory tests revealed a normal white blood cell (WBC) count but detected neutrophilia (WBC count, 8.36 × 10^3^/*μ*L; neutrophils, 96.5%), hyponatremia (Na, 128 mEq/L), a markedly increased creatine kinase (CK) (3254 IU/L), and increased fibrinogen/fibrin degradation products (FDP) (31.2 *μ*g/mL). Chest CT revealed extensive patchy shadows mainly in the right upper lung lobe. Based on these findings, an intravenous broad-spectrum antibiotic (tazobactam/piperacillin, 18 g/day) was initiated.

On Day 2, a* Legionella* urinary antigen test was positive for* Legionella pneumophila* serogroup 1, suggesting a* Legionnaire pneumophila* infection. The patient's clinical presentation and rapid progression of respiratory failure supported this diagnosis. Thus, intravenous levofloxacin (500 mg/day) and azithromycin (500 mg/day) were initiated.

On Day 3, the patient required a respirator, and low-dose hydrocortisone and sivelestat sodium hydrate, a selective neutrophil elastase inhibitor, were initiated. The patient's respiratory condition gradually improved, and he was extubated on Day 17.

**Figure 1:**
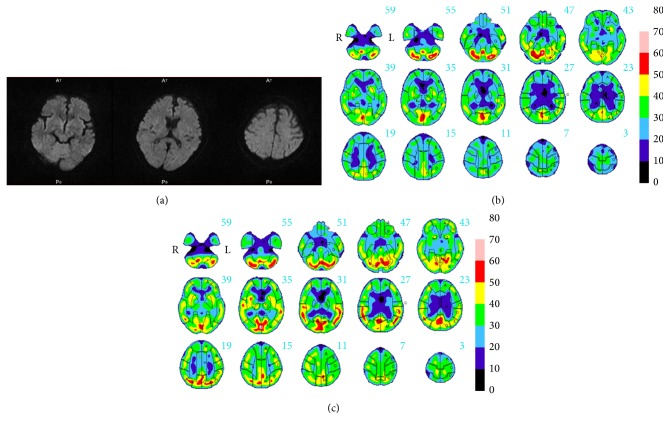
(a) Axial diffusion-weighted magnetic resonance images of the brain in Case 1 obtained on Day 22, revealing no abnormalities. (b) Single-photon emission computed tomography (SPECT) images of the brain using technetium-99m-ethyl cysteinate dimer, analyzed with a 3-dimensional stereotactic region of interest template (3DSRT), in Case 1 obtained on Day 23, demonstrating multifocal hypoperfusion mainly in the frontal and temporal lobes. (c) Corresponding SPECT images of the same patient obtained on Day 65, showing improvement in hypoperfusion areas to almost normal levels.

However, even after the cessation of sedatives, the patient demonstrated a variety of neurologic symptoms. He was awake but was disoriented, was dysthymic, and could not speak. He was able to lift his upper limbs but exhibited muscle weakness in his right upper and lower limbs. He could not move his limbs smoothly and showed a lack of coordination in the finger-to-nose test. Subsequent MRI of the brain revealed no abnormalities (Figure 1(a)). Cerebrospinal fluid (CSF) analysis revealed that the CSF cell counts, protein and glucose concentrations, and immunoglobulin G index were all normal. Gram staining and bacterial culture of the CSF were also negative. One week after the initial examination, the patient exhibited improvements in limb movement, but his disorientation remained. Electroencephalography showed diffuse slow activity. SPECT of the brain using a technetium-99m-ethyl cysteinate dimer (99mTc-ECD) was performed on Day 23 and showed multifocal hypoperfusion mainly in the frontal and temporal lobes (Figure 1(b)). Thereafter, his consciousness gradually became clearer without any treatment, and he was able to speak simple words. Six weeks after the initial examination, a second SPECT scan was obtained and revealed that the hypoperfusion in the frontal and temporal lobes had improved to almost normal levels (Figure 1(c)). At that time, laboratory tests revealed that most values, including FDP, had returned to normal limits. He recovered the strength in all 4 extremities by Day 60, and the patient was able to walk and speak falteringly when he was discharged on Day 68.

### 2.2. Case 2

A 59-year-old man was admitted to our hospital for difficulty in breathing. He had a medical history of subdural hematoma caused by an accident but had been well without any neurologic abnormalities. He lived in the same dormitory and used the same bathroom as the patient in Case 1. He did not drink alcohol regularly but had a 20 pack-year history of smoking. He had developed a cough 2 days before admission and had experienced a headache and tremors the following day.

On admission (Day 1), his body temperature was 36.9°C, and chest auscultation revealed normal respiratory sounds. He was confused, and neurologic examination revealed disorientation and bilateral intention tremor. Laboratory tests revealed an increased WBC count (20.19 × 10^3^/*μ*L), increased C-reactive protein (21.16 mg/dL), mild hyponatremia (Na, 132 mEq/L), mild liver dysfunction (aspartate aminotransferase, 59 IU/L; alanine aminotransferase, 54 IU/L), mildly increased CK (240 IU/L), and an increased D-dimer (5.12 *μ*g/mL). Chest radiography and CT showed left lower lobe consolidation. A* Legionella* urinary antigen test was positive for* Legionella pneumophila* serogroup 1. Thus, the patient was diagnosed as having Legionnaires' disease, and intravenous levofloxacin (500 mg/day) and azithromycin (500 mg/day) were initiated.

On Day 10, the patient's chest radiography showed improvement, and he was afebrile. However, his consciousness was still impaired, and his Mini-Mental State Examination (MMSE) score was 19/30. Brain CT showed a left subdural hematoma with a mild mass effect and a pituitary tumor, but acute lesions were not observed. Thus, we suspected that his neurologic symptoms were caused by the* Legionella* infection.

**Figure 2:**
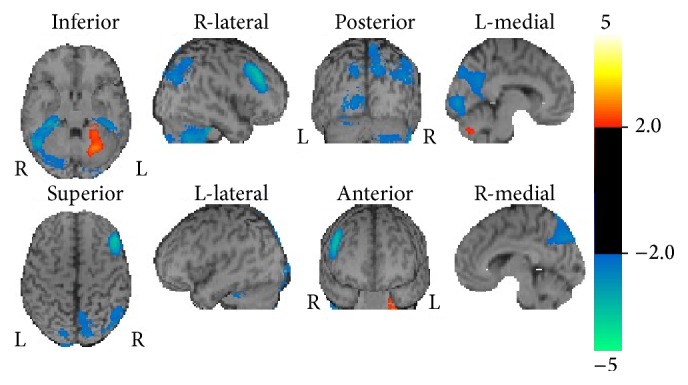
Single-photon emission computed tomography images of the brain using technetium-99m-ethyl cysteinate dimer, analyzed with the *Z*-score imaging system (eZIS), in Case 2 obtained on Day 13, showing multifocal hypoperfusion mainly in the cortex of the parietal lobe, the precuneus, the right cerebellum, and the posterior cingulate cortex.

On Day 13, SPECT of the brain using 99mTc-ECD revealed multifocal hypoperfusion mainly in the parietal, precuneus, and posterior cingulate cortices and in the right cerebellum (Figure 2). His symptoms gradually improved without any treatment, and his MMSE score increased to 23/30. The patient had mild disorientation and slurred speech when he was discharged on Day 24.

## 3. Discussion

Legionnaires' disease is commonly associated with neurologic symptoms. Cerebral symptoms include confusion and dysarthria while cerebellar symptoms include gait and limb ataxia. Even in the presence of neurologic symptoms, most cases lack CNS abnormalities on neuroimaging studies, suggesting that CT and brain scintigraphy are not sufficiently sensitive for detecting brain lesions in patients with Legionnaires' disease.

Although brain MRI seldom detects abnormalities associated with* Legionella* infection, recent studies have reported the detection of transient lesions associated with this disease via MRI. In a report of 2 patients who developed acute disseminated encephalomyelitis, brain MRI detected multiple disseminated lesions [5]. Similarly, in other reports of patients who presented with cerebral and cerebellar symptoms, brain MRI revealed reversible lesions associated with Legionnaires' disease in the splenium of the corpus callosum (SCC), which resolved with clinical improvement within 2 weeks [6, 7]. However, there were the limitations of MRI for detecting brain lesions in Legionnaires' disease. In a patient with memory loss, disorientation, cerebellar dysfunction, hypothalamic dysfunction, and mild hyponatremia, Imai et al. observed that diffusion-weighted MRI of the brain performed 2 days after admission showed marked hyperintensity in the SCC. Nevertheless, SPECT performed 6 days after admission showed cerebellar and frontal lobe hypoperfusion that was not observed on the corresponding MRI. Interestingly, MRI performed 14 days after the initial study showed complete resolution of the SCC, whereas SPECT performed 45 days after the initial study showed that hypoperfusion was still present in the brain [4]. Similarly, in a patient who developed acute cerebellar ataxia after infection, SPECT performed on the 15th day of illness revealed marked cerebellar hyperperfusion, even though contrast-enhanced CT and MRI scans of the brain were normal [8]. Collectively, these reports suggest that SPECT is capable of detecting the prolonged effects of* Legionella* infection in the CNS, in contrast with the rapid resolution of a CNS lesion observed on MRI.

In our cases, all neuroimaging tests were performed after Day 13. At that time, the patients' cerebellar dysfunction had mostly disappeared but not their cerebral dysfunction. These facts might explain why MRI failed to demonstrate any abnormalities, and SPECT revealed cerebral lesions but not cerebellar lesions.

Hypoperfusion of the cerebral and cerebellar regions on SPECT is not specific findings of Legionnaires' disease. It was reported previously that five children with acute cerebellar ataxia after viral infection showed decreased cerebellar blood flow on SPECT [9]; an infant patient showed hypoperfusion of the bilateral hippocampal regions and the unilateral temporal region on SPECT after viral infection [10]. Hypoperfusion of the cerebral regions on SPECT after severe infection was reported mostly in infant patients, and most cases of them were caused by viral infection. Adult patients showed that prolonged hypoperfusion of brain on SPECT after bacterial infection was relatively rare.* Legionella* spp. might be likely the cause of hypoperfusion of brain.

The mechanisms that cause neurologic dysfunction in patients with Legionnaires' disease remain unclear. Results of CSF analysis are usually normal. In a previous study, autopsies of 40 patients with* Legionella pneumophila* pneumonia, 16 of whom had neurologic symptoms, revealed no evidence of infection attributable to a* Legionella* species [11]. Therefore, it was speculated that the mechanisms that cause* Legionella*-induced neurologic dysfunction may involve production of a neurotoxin-like substance or immune-mediated mechanism. Although no* Legionella*-produced neurotoxin has been found, a protease from* Legionella pneumophila* could cause pulmonary damage in the absence of bacterial cells [12]. Studies of “peripheral” nerves involved in Legionnaires' disease revealed lesions similar to those induced by toxic insults [1]. These data support the neurotoxin hypothesis.* Legionella* spp. can modulate apoptotic pathways and disrupt cell membranes intracellularly, leading to cell death by secreted effectors [13]. These effectors and other virulence factors, such as heat shock proteins, phospholipase, and liposaccharides, might work as a neurotoxin intercellularly. Additionally, the discordance between findings obtained by SPECT and MRI suggests a contribution of vasculopathy to Legionnaires' disease. It was previously reported that patients with CNS lupus also showed abnormal cerebellar blood flow on SPECT, despite the absence of any abnormality on MRI [14]. This suggests the possibility that neurologic dysfunction in Legionnaires' disease might be caused by an immune-mediated mechanism that is similar to CNS lupus.

## 4. Conclusion

We experienced 2 cases of* Legionella pneumophila* pneumonia with prolonged neurologic symptoms. In both cases, SPECT was able to detect hypoperfusion in the brain, even though no abnormalities were observed on CT or MRI. Vasculopathy detected by SPECT might be one of the causes of neurologic dysfunction in patients with Legionnaires' disease.
